# Generating universal anti-CD19 CAR T cells with a defined memory phenotype by CRISPR/Cas9 editing and safety evaluation of the transcriptome

**DOI:** 10.3389/fimmu.2024.1401683

**Published:** 2024-05-29

**Authors:** Kristina Pavlovic, MDolores Carmona-Luque, Giulia I. Corsi, Noelia Maldonado-Pérez, Francisco J. Molina-Estevez, Esther Peralbo-Santaella, Marina Cortijo-Gutiérrez, Pedro Justicia-Lirio, María Tristán-Manzano, Víctor Ronco-Díaz, Antonio Ballesteros-Ribelles, Alejandro Millán-López, Paula Heredia-Velázquez, Carla Fuster-García, Toni Cathomen, Stefan E. Seemann, Jan Gorodkin, Francisco Martin, Concha Herrera, Karim Benabdellah

**Affiliations:** ^1^ Department of Genomic Medicine, Pfizer-University of Granada-Andalusian Regional Government Centre for Genomics and Oncological Research (GENYO), Granada, Spain; ^2^ Cell Therapy Group, Maimonides Institute of Biomedical Research in Cordoba (IMIBIC), Cordoba, Spain; ^3^ Department of Veterinary and Animal Sciences, Center for non-coding RNA in Technology and Health, University of Copenhagen, Thorvaldsensvej, Denmark; ^4^ Flow Cytometry Unit, Maimonides Biomedical Research Institute of Cordoba (IMIBIC), Cordoba, Spain; ^5^ LentiStem Biotech, Pfizer-University of Granada-Andalusian Regional Government Centre for Genomics and Oncological Research (GENYO), Granada, Spain; ^6^ Department of Human Anatomy and Embryology, Faculty of Medicine, University of Granada, Granada, Spain; ^7^ Institute for Transfusion Medicine and Gene Therapy, Medical Center - University of Freiburg, Center for Chronic Immunodeficiency (CCI), Medical Center - University of Freiburg, Faculty of Medicine, University of Freiburg, Freiburg, Germany; ^8^ Department of Biochemistry and Molecular Biology III and Immunology, Faculty of Medicine, University of Granada, Granada, Spain; ^9^ Biosanitary Research Institute of Granada (ibs.GRANADA), University of Granada, Granada, Spain; ^10^ Department of Hematology, Reina Sofia University Hospital, Cordoba, Spain; ^11^ Department of Medical and Surgical Sciences, School of Medicine, University of Cordoba, Cordoba, Spain

**Keywords:** anti CD 19 CAR-T cells, CRISPR/Cas9, allogeneic CAR-T cells, memory CAR-T cells, CRISPRroots

## Abstract

**Introduction:**

Chimeric antigen receptor-expressing T cells (CAR T cells) have revolutionized cancer treatment, particularly in B cell malignancies. However, the use of autologous T cells for CAR T therapy presents several limitations, including high costs, variable efficacy, and adverse effects linked to cell phenotype.

**Methods:**

To overcome these challenges, we developed a strategy to generate universal and safe anti-CD19 CAR T cells with a defined memory phenotype. Our approach utilizes CRISPR/Cas9 technology to target and eliminate the *B2M* and *TRAC* genes, reducing graft-versus-host and host-versus-graft responses. Additionally, we selected less differentiated T cells to improve the stability and persistence of the universal CAR T cells. The safety of this method was assessed using our CRISPRroots transcriptome analysis pipeline, which ensures successful gene knockout and the absence of unintended off-target effects on gene expression or transcriptome sequence.

**Results:**

*In vitro* experiments demonstrated the successful generation of functional universal CAR T cells. These cells exhibited potent lytic activity against tumor cells and a reduced cytokine secretion profile. The CRISPRroots analysis confirmed effective gene knockout and no unintended off-target effects, validating it as a pioneering tool for on/off-target and transcriptome analysis in genome editing experiments.

**Discussion:**

Our findings establish a robust pipeline for manufacturing safe, universal CAR T cells with a favorable memory phenotype. This approach has the potential to address the current limitations of autologous CAR T cell therapy, offering a more stable and persistent treatment option with reduced adverse effects. The use of CRISPRroots enhances the reliability and safety of gene editing in the development of CAR T cell therapies.

**Conclusion:**

We have developed a potent and reliable method for producing universal CAR T cells with a defined memory phenotype, demonstrating both efficacy and safety *in vitro*. This innovative approach could significantly improve the therapeutic landscape for patients with B cell malignancies.

## 1 Introduction

Chimeric antigen receptor (CAR) T cell therapy has revolutionized the immunotherapy field during the last years, offering a promising approach for treating different conditions. These cells are standard T lymphocytes that have been genetically modified to express in their membrane the CAR construct. This receptor has a characteristic structure, presenting an extracellular binding domain followed by a hinge region that connects with an intracellular signaling domain which contains a CD3ζ region and one or two costimulatory domains (depending on the CAR generation), generally CD28 and/or 4-1BB (1–4).

The impressive results on hematological malignancies have led to the approval of six CAR-T cell products by the European and American agencies (EMA and FDA respectively) for the treatment of B-cell acute lymphocytic leukemia (B-ALL), non-Hodgkin lymphoma (NHL) and multiple myeloma (MM). All these products are based on autologous CAR T cells, and this comes with several limitations, mainly due to the time and cost of the manufacturing process, which can take up to 3 weeks (5). In some cases, the time of the administration is vital for the success of the procedure, as many patients succumb to their disease and/or develop significant complications prior to receiving their CAR product (6, 7). Also, the quantity and more importantly quality of the starting material, which in this case are T cells from patients that have previously received several treatments, is of utmost importance for the efficacy of this therapy. During the Kymriah pivotal trial, 9% of the enrolled patients could not receive the product due to manufacturing failure (8). Additionally, terminally differentiated CAR-T phenotype due to disease and previous treatment significantly hampers the possibility of exerting long-lasting antitumor effects (9).

To overcome the above specified limitations associated with patients’ cell features, and to standardize a more cost-effective CAR T production, several strategies have been set up to generate alternatives, primarily based on allogeneic products (5, 10, 11). Although there are several allogeneic cell sources (12–14), T cells derived from peripheral blood mononuclear cells (PBMCs) from healthy individuals are the most widely used.

However, such T cells generate allogeneic HLA-mismatched recognition leading to graft-versus-host disease (GVHD) and/or host-versus-graft (HVG) immune rejection, resulting in diminished persistence and decreased effectiveness against tumor cells (15–17).

Gene editing tools have offered the possibility of eliminating the expression of genes involved in this rejection. Both T cells receptor (TCR) and Human Leukocyte Antigen (HLA) I or II, or associated genes can be successfully knocked out, generating universal CAR-T cells. In 2012, a seminal study by Torikai et al. described for the first time the possibility of eliminating TCR using Zinc-Finger nucleases (ZFNs) (18), and that the elimination of TCR expression does not weaken the antitumor properties of CAR-T cells. The same approach was subsequently validated using CRISPR technologies, paving the way for the generation of easy protocols for the creation of allogeneic universal CAR-T (19). The elimination of TCR also benefits immunotherapy approaches based on cancer-specific TCR surface expression, in which the presence of both endogenous TCRs and cancer-specific CARs may induce the development of heterodimers which, in turn, may diminish the efficacy of the therapy (20). As with TCR, several authors have successfully used genome editing technologies to knock out the B2-microglobulin (B2M). B2M protein forms a heterodimer with HLA class I proteins and therefore the suppression of the B2M gene would induce a depletion of all the HLA I molecules (HLA-A, -B, -C, -E, -F, and -G). Lee et al, have successfully edited primary human T cells for HLA-I, and the authors report phenotypically unaltered and functionally intact T cells (21). Based on the efficiency and safety profile of genome-edited CAR-T cells, several clinical trials are ongoing to analyze safety and efficacy of universal CAR-T cells eliminating TCRα alone or in combination with other genes (NCT02808442, NCT02746952, NCT02735083, NCT03166878; NCT03229876, NCT03545815, NCT03398967, NCT03666000).

Current tools for predicting gRNA safety are often focused on analyzing predicted off-target cuts, providing a limited perspective on their impact on gene expression and possibly overlooking critical off-targets. To address this limitation, we developed the CRISPRroots approach that combines CRISPR–Cas9-mediated edits with RNA-Seq data analysis (22). CRISPRroots integrates guide RNA binding properties, gene expression changes, and sequence variants to identify and prioritize potential off-target sites that affect the transcriptome and that may impact expression. This allows a more accurate identification of potential safety issues associated to the GE protocol (22).

A second concern in the field is the failure of these edited cells to expand and maintain sufficient levels of engraftment in patients (23). Prolonged therapeutic responses have been associated with the sustained presence of CAR T cells and robust T cell proliferation (24–26). Notably, the work of Fraietta et al. (27) identified CAR T cell fitness as a predictor of therapeutic success. Additionally, repeated exposures to tumoral antigens lead to exhaustion and therefore failure of the cells (28). Also, intrinsic factors, such as cell differentiation state, significantly influence cell fitness, and most favorable clinical results correlate with a higher percentage of memory cells in the final CAR product (27, 29). Based on this data we propose using universal CAR T cells with a predefined memory phenotype.

In 2011, the existence of a stem cell memory (Tscm) compartment was discovered (30). These cells possess increased self-renewal capabilities and the unique ability to generate diverse memory and effector T cell subsets *in vitro*. Besides, the transfer of autologous CAR-T cells enriched in T stem-like cells resulted in better control of established tumors and resistance to tumor rechallenge (29). Several recent procedures have evolved over the last years in order to produce CAR-T enriched in Tscm and/or Tcm, based on several strategies such as the inclusion of specific homeostatic cytokines like IL-7 or IL-15 (31), the use of Wnt agonists that inhibit human memory CD8 T cell differentiation (32) or by metabolic intervention (33).

Therapy outcomes are unpredictable when the infused product has an arbitrary CD4:CD8 proportion, which additionally is always different depending on the T cell source. It has been long described that CAR T cells derived from CD4 naïve and memory compartments exhibit increased *in vivo* persistence and antitumoral activity. It has also been described that CD8 memory cells are critical for CAR T cells survival (5). Therefore, maintaining a defined proportion of CD4 and CD8 in CAR-T cell products is crucial for sustaining antitumor responses over the long term (34).

In this manuscript, besides evaluating viability and safety of universal CAR T products, as novelty in the field, we propose the generation of universal CAR-T cells with a defined memory phenotype. We scrutinized various cellular products, exploring scenarios with predefined CD4:CD8 ratios while adjusting the presence of Tscm and Tcm cells in each subset. Our final approach involved precision targeting with CRISPR/Cas9 to simultaneously eliminate the B2M and TRAC genes in CAR T cells, application of the CRISPRroots pipeline on RNA-seq data to substantiate product safety, and implementation of a purification step to enrich T memory cells.

## 2 Materials and methods

### 2.1 Human samples and cells lines

Peripheral blood mononuclear cells (PBMCs) were obtained from apheresis products derived from healthy donors at the Hematology Department of the Hospital Universitario Reina Sofía (Córdoba, Spain). Written informed consent was obtained from all donors. Primary T cells were isolated using the Pan T cell Isolation Kit (Miltenyi Biotec) and further separated using the AutoMACS Pro Separator (Miltenyi Biotec) through negative selection. Following isolation, T cells were activated with Human T cell Transact (Miltenyi Biotec) and cultured in TexMACS (Miltenyi Biotec) medium supplemented with 10 ng/ul of IL7 and IL15 (Miltenyi Biotec). The cell culture was conducted in GRex 24-well plates (ScaleReady).

Namalwa (ATCC CRL-1432) and Jurkat (ATCC TIB-152) cells were cultured in RPMI 1640 Medium (Biowest) medium supplemented with 10% Fetal Bovine Serum (FBS) (Lonza) and 1% penicillin/streptomycin (Biowest). HEK293T cells (ATCC CRL-11268) were cultured in DMEM (Biowest) medium supplemented with 10% FBS and 1% penicillin/streptomycin. Cells were routinely tested for mycoplasma.

Namalwa cells were genetically modified at our lab to express enhanced green fluorescent protein (eGFP) and Nanoluciferase (NanoLuc) using SEWP lentiviral vectors (35). Additionally, a Namalwa eGFP-NanoLuc CD19- cell line was created by knocking out the CD19 antigen. This cell line served as negative non-specific control for cytotoxicity assays.

For isolation of Natural Killer (NK) cells, a sample of peripheral blood was collected from a healthy donor, and NK cells were isolated directly using the RosetteSep™ Human NK Cell Enrichment Cocktail kit (STEMCELL), following the manufacturer’s protocol. A SepMate™ PBMC Isolation Tube with a total blood volume of 15 mL was utilized. The obtained fraction yielded approximately 3x10^6^ cells, which were seeded in RPMI supplemented with 10% male human AB serum (LINUS) and Human IL-2 IS (1000 U) (Miltenyi Biotec) in a 24-well G-REX plate. Cells were cultured for 5 days before performing the assays.

### 2.2 CAR construction and lentiviral production

Creative Biolabs supplied a third-generation anti-CD19 CAR (clone FCM63, αCD19-28-BB-zz) which incorporates the intracellular domains of CD28, 4-1BB and CD3z, along with a truncated EGFR as a selection marker. Lentiviral vectors carrying this CAR were manufactured in our lab by co-transfection of HEK-293T cells with the plasmid of interest, the plasmid pCMVDR8.91 and the p-MD-G plasmid as previously described by our team (36).

The supernatants from the resulting culture were collected after 48 and 72 hours and concentrated by centrifugation at 90.000g for 2 hours at 4°C. The resulting viral particles were resuspended in TexMACS and stored at -80°C. To determine the viral titer (transducing units per ml, TU/ml), 10^5^ Jurkat cells were transduced with the obtained lentiviral particles by spinoculation, at 800g for 30 min at 32°C. After 48h of culture, the percentage of positive cells was assessed by flow cytometry and the following formula was applied:


$$\frac{TU}{ml}=\frac{\left[N\;of\;transduced\;cells\;x\;\%\;of\;positive\;cells\;\right]}{\;ml\;virus}$$

### 2.3 Transduction and electroporation of T cells.

To generate universal CAR T cells, CD3+ isolated cells were stimulated 24h prior to transduction with the viral vectors carrying the CAR construction at a multiplicity of infection (MOI) of 5. Transduction was carried out as mentioned above, but with a spinoculation time of one hour. At 48-72 hours post-transduction, *TRAC* and *B2M* loci were simultaneously targeted with the CRISPR/Cas9 editing system (sgRNAB2M= acucacgcuggauagccucc and sgRNATRAC: ucaggguucuggauaucugu, purchased in GenScript). For this simultaneous delivery of single guide RNAs (sgRNA), ribonucleoparticles (RNP) were generated by incubating the sgRNAs with the Streptococcus pyogenes Cas9 nuclease (Integrated DNA Technologies) at 37°C for 20 minutes, in molar ratio 2.4:1 (each sgRNA 1.2:1). We used the Amaxa 4D-Nucleofector (Lonza) following the commercial protocol to electroporate the RNP complex into T cells. Media was renewed after 5h in both transduction and nucleofection protocols.

The knockout efficiency was assessed by two methods: flow cytometry was conducted at different time-points after editing, to measure expression of CD3 and HLA-I in cells surface, and Sanger sequencing was performed 7 days after editing, followed by Interference CRISPR Edits (ICE) to evaluate the efficacy of “on-target” modifications, specifically by analyzing the number of insertions and deletions (indels) generated by the CRISPR/Cas9 system. For Sanger sequencing we first performed an amplification by PCR using the following primers: FwTRAC: TTGATAGCTTGTGCCTGTCC, RvTRAC: GAATAATGCTGTTGTTGAAGGC, FwB2M: GGCACGCGTTTAATATAAGT and RvB2M: GAGGCACAGTACATCTTGGA. These primer sets were used to amplify the respective target regions for subsequent Sanger sequencing analysis.

### 2.4 Analysis of RNA-seq data from CRISPR/Cas9 edited cells

RNA extraction from cells harvested 7 days post editing is performed followed by sequencing analysis. Paired-end Poly(A) RNA sequencing data of edited (knockout) and non-edited (wildtype, WT) samples, each with four technical replicates, were analyzed using the CRISPRroots pipeline v.1.3 (22). Reference files, including human genome hg38, GENCODE primary assembly annotations v. 43 (37), and ribosomal RNA sequences from SILVA v. 119 (38), were downloaded along with the CRISPRroots software (https://rth.dk/resources/crispr/crisprroots/). Reads were processed with Cutadapt v.2.10 (39) to trim adapters, low-quality ends, and flanking “N” bases. Variations of the reference genome discovered in the transcriptome of non-edited samples were considered in the analysis to search for potential off-targets. The sgRNAs for B2M and TRAC were provided to the pipeline for the analysis of on- and off-target edits. Batch effects in DESeq2 were accounted for (40), and the batch effect was corrected in the Principal Component Analysis using the function remove Batch Effect from limma v.3.52.4 (41) on the regularized logarithm transformed read counts in R v.4.3. 0 (42). Overrepresentation analysis of terms related to significantly differentially expressed genes (DEGs) was conducted in stringApp v.2.0.1 (43), and terms with redundant enriched genes were filtered out. The display of enriched terms was generated with enrichment-visualizer (https://github.com/gcorsi/enrichment-visualizer).

After obtaining a list of putative off-target edits, we designed primers flanking their regions and performed Sanger sequencing with further ICE analysis to evaluate the existence of indels.

### 2.5 Translocation quantification by ddPCR

Putative translocation reference sequences (refseqs) were generated *in silico* based on Homo sapiens chromosome 14 (GRCh38.p14 Primary Assembly) and Chromosome 15 (GRCh38.p14 Primary Assembly) public sequences using SnapGene v5.2 Software.

Primers and probes covering a 300 bp window from the CRISPR/Cas9 cutting sites were engineered using NCBI online tools (https://www.ncbi.nlm.nih.gov/tools/primer-blast/).

For ddPCR loci quantification, primers and probes were mixed in BioRad ddPCR Supermix (no dUTP) according to manufacturer instructions. In each reaction 22 µl of the following mix were prepared: 11 µl of 2X Super mix, 1,1 µl of 20X primers/probe mix and 2.2 µl of a 10 ng/µl DNA solution. Then 20 µl were placed into the DG8 cartridges (BioRad) in a QX200 droplet generator (BioRad) using droplet generator oil for probes (BioRad). Once droplets were ready, 40 µl were transferred and sealed in a PCR plate for amplification according to manufacturer recommendations. All PCRs were run in a C1000Touch thermocycler (BioRad) and acquired in a QX200 droplet reader (BioRad). Data analysis was performed using the provided QuantaSoft version 1.7.4 software (BioRad).

### 2.6 Cytotoxicity assay

For cytotoxicity assays, Namalwa eGFP-NanoLuc cells were seeded in co-culture with effector CAR T cells, at a 15:1 target to effector ratio. TexMACS media without supplements was used and the co-culture was incubated for 72 hours. To assess lysis over different tumor encounters, additional Namalwa eGFP-NanoLuc cells were added to the culture every 72 hours to restimulate the cells. Specifically, the number of plated Namalwa cells was doubled after the second rechallenge, and this pattern continued every 72 hours thereafter.

In parallel, the same was done with Namalwa eGFP-NanoLuc CD19KO to calculate specific lysis based on the following formula. In this case, no rechallenges were performed.


$$\%Specific\;lysis=\left(1-\frac{\left(\frac{\%CD19^{+}in\;CAR^{+}population}{\%CD19^{-}in\;CAR^{+}\;population}\right)}{\left(\frac{\%CD19^{+}in\;CAR^{-}population}{\%CD19^{-}in\;CAR^{-}\;population}\right)}\right)x100$$

These results were evaluated using flow cytometry, specifically by tracking the loss of eGFP fluorescence as a result of cell lysis. By monitoring the decrease in eGFP fluorescence over time, we can assess the level of cell lysis induced by the effector CAR T cells and determine the cytotoxic activity against Namalwa eGFP-NanoLuc cells. Additionally, total cell counts of T and Namalwa cells were evaluated using CountBright™ Absolute Counting Beads (ThermoFisher).

### 2.7 Alloreactive response

To evaluate the response against allogeneic cells, we conducted a mixed lymphocyte reaction (MLR) using the edited cells and PBMCs from another healthy donor at a 1:1 ratio. For tracking cell division of each pool, non-edited and edited T cells were labelled with Carboxyfluorescein succinimidyl ester (CFSE; Molecular Probes, Invitrogen), and allogeneic PBMCs with Cell Trace Violet (CTV; Invitrogen), following the manufacturer protocol. This co-culture was maintained for 6 days in TexMACS with no supplements. Loss of CFSE or CTV was analyzed by flow cytometry, and it was directly related to proliferation of each pool of cells.

In addition, to evaluate proliferation, we measured the activation marker CD25 by staining the cells and further analyzing it through flow cytometry. Furthermore, we measured the secretion of interferon-gamma (IFNγ) as an indicator of immune response using an enzyme-linked immunosorbent assay (ELISA, Biolegend).

For determining whether NK cells would attack T cells with no HLA-I complex, we also conducted a MLR assay with our edited and non-edited CAR cells versus NK cells from a healthy donor, at a 1:1 ratio. For tracking the T cells in the co-culture we labelled them with CFSE, following the manufacturer protocol. The co-culture was maintained for 24h in RMPI with no supplements, and results were analyzed by flow cytometry.

### 2.8 Flow cytometry and cell sorting

A flow cytometry analysis was performed for assessing the phenotype and exhaustion stage of the cellular products resulted from the allogeneic and cytotoxic assays. For human T cells phenotyping, the following anti-human monoclonal antibodies (mAbs) were used: CD3, CD45RA, CD2, CD4, CD8, EGFR and HLA-ABC. T cells alloreactive response was quantified with the CD25 mAb, and cellular exhaustion with the TIM3, PD1 and LAG3 mAbs. Samples were acquired on a FACSVerse cytometer (BD Biosciences, USA) in GENYO (Granada, Spain).

The cellular product (bulk and subsets) immunophenotype was assessed on day four and day eleven after the edition. The CD4+ and CD8+ T cells subsets naïve (Tn) CD45RA+/CD45RO-/CCR7+/CD95-, stem cell memory (Tscm) CD45RA+/CD45RO-/CCR7+/CD95+, central memory (Tcm) CD45RA-/CD45RO+/CCR7+, and effector memory (Tem) CD45RA-/CD45RO+/CCR7- was quantified using the following anti-human mAbs: CD3, CD4, CD8, CD45RA, CD45RO, CCR7 and CD95. Samples were acquired in a LSRFortessa SORP flow cytometer (Becton Dickinson) located in the facilities of the Flow Cytometry Research Support Service at IMIBIC (Cordoba, Spain). For more mAbs information, see **Supplementary Table 1**.

At least 10.000 events were analyzed for each marker. FlowJo v10 software was used for the analysis of the resulted cytometry files.

The CD4+ and CD8+ Tscm and Tcm subsets were sorted to >80% enrichment by fluorescence-activated cell sorting (FACS) on a FACSAria III (Becton Dickinson) in IMIBIC (Córdoba, Spain). The design of the conjugated-mAbs panel was the same as that used for flow cytometry. After the cell sorting procedure, a quality control was performed to check the percentage of enrichment of isolated subsets.

### 2.9 T cell subsets co-culture and characterization

After cell sorting, enriched CD4+ and CD8+ Tscm subsets, and CD4+ and CD8+ Tcm subsets were co-cultured at a 1:1 CD4+:CD8+ ratio, performing different subsets combinations such as CD4+Tscm: CD8+Tscm, CD4+Tscm: CD8+Tcm, and CD4+Tcm: CD8+Tscm. Co-cultures were maintained for seven days in TexMACS (Miltenyi Biotec) medium supplemented with 10 ng/ul of IL7 and IL15 (Miltenyi Biotec). The cell culture was conducted in GRex 24-well plates (ScaleReady).

### 2.10 Cytokine secretion

To evaluate the cytokine production of the edited and sorted CAR T cells following tumoral stimulation, we co-cultured 5×10^4^ effector T cells with Namalwa target cells at a 15:1 target to effector ratio. After 72 hours of co-culture, supernatants were collected and frozen at -80°C. The levels of TNFα and IFNγ were measured using the ab100654-TNF alpha Human ELISA Kit (Abcam^®^, Cambridge, CB2 0AX, UK) and ab174443-Human IFN-gamma SimpleStep ELISA^®^ Kit (Abcam^®^, Cambridge, CB2 0AX, UK), respectively. The measurements were performed following the manufacturer’s instructions for each kit.

### 2.11 Data analysis

Figures were generated using the GraphPad Prism 9 software and the Matplotlib library in Python3 (44). The data are represented as the mean ± standard error of the mean (SEM). Statistics were conducted using the same software, and each test is indicated in the figure legends.

## 3 Results

### 3.1 Simultaneous targeting of *TRAC* and *B2M* loci efficiently reduces expression of TCR and HLA-I in T cells without altering their phenotype

**Figure 1:**
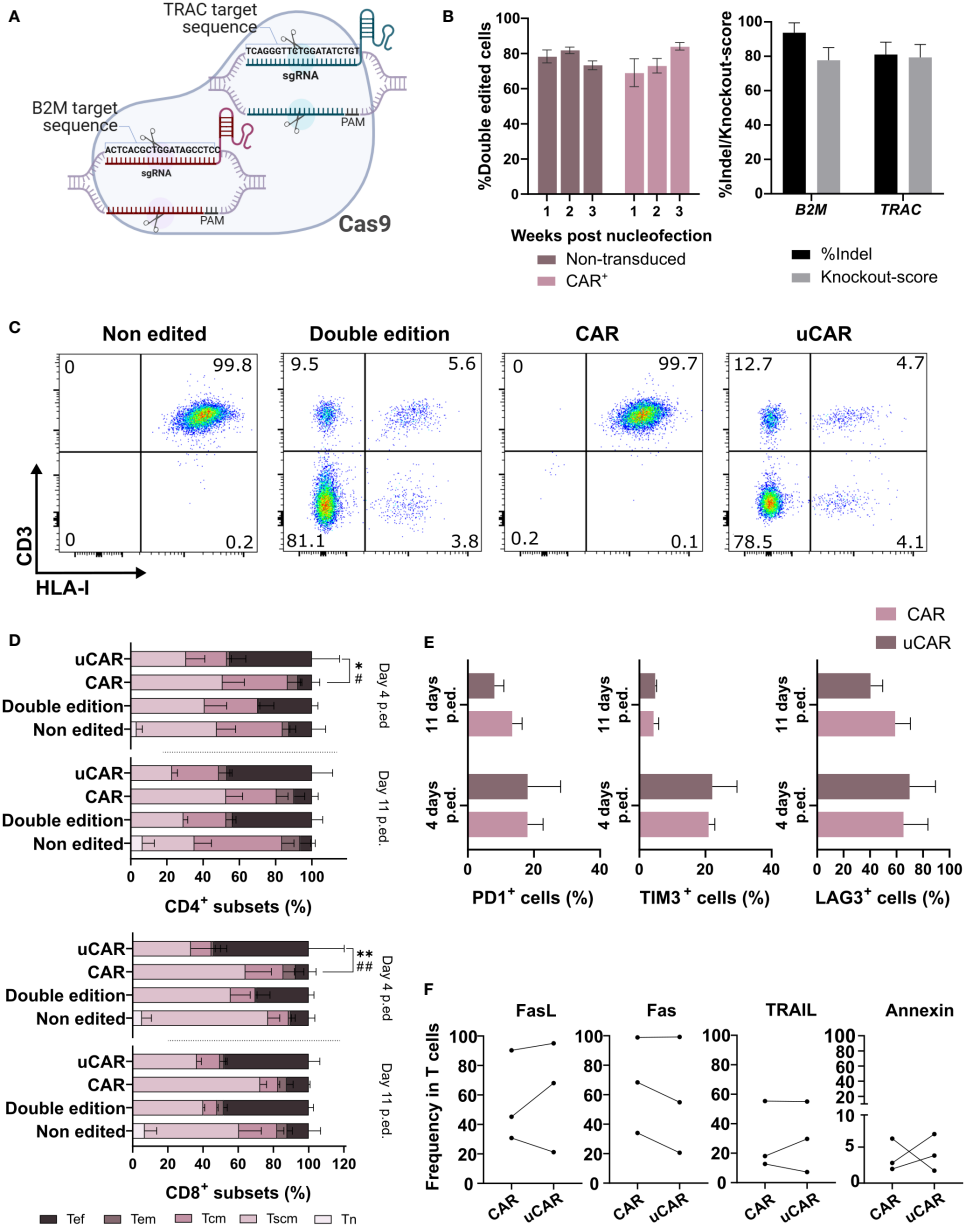
Phenotypical characterization of T cells after edition showed no differences between unedited, edited and transduced T cells. **(A)** Ribonucleoparticle used for edition of T and CAR T cells. Two different sgRNAs were incubated with the Cas9 in order to form one complex targeting both loci. Created with BioRender.com. **(B)** In the left graph are represented CAR^-^ (Non-transduced) and CAR^+^ (transduced) cells that did not present neither HLA-I nor CD3 in their surface. N=10. In the right graph, insertions and deletions (indels) generated in the target sites of the RNP. N=3. **(C)** Cytometry analysis of the different conditions, showing expression of HLA-I (x axis) and CD3 (y axis) in cells surface. The cytometry data presented herein are stochastic outcomes, reflecting the most reliable approximation of the underlying reality of the results. **(D)** Representation of the frequencies of the different T cells memory subsets (naïve, Tn; stem cell memory, Tscm; central memory, Tcm; and effector memory, Tem) and effector cells (Tef) 4 and 11 days after the edition was performed. Statistical differences were represented only between CAR and uCAR conditions, which we found of more importance. Sign * indicates differences between Tscm groups, and # indicates differences between Tef groups. N=3. **(E)** Graph showing frequency in cells of triple positive cells for the exhaustion markers PD1+, LAG3+ and TIM3. N=4. **(F)** Expression of activation induced cell death (AICD) markers (FasL, Fas, TRAIL) and annexin V in CAR and uCAR T cells. N=3; Friedman and Wilcoxon test were performed in comparisons. **p< 0.01. Non-significant results were not labelled in the graphs. *p<0.05, **p< 0.01, #p<0.05, ##p<0.01.

Several strategies can be used for ablation of gene expression in T cells, with the aim of generating universal CAR T cells (uCAR T). Here, we investigated the efficacy and safety of simultaneous elimination of *TRAC* and *B2M* genes in T cells through electroporation with mixed Cas9/sgRNA RNPs (**Figure 1A**). As evident from flow cytometric analysis, elimination of the expression of both genes was achieved in over 80% of T cells and CAR T cells (**Figures 1B** left and **1C**). Interference CRISPR Edits (ICE) analysis confirmed that around 85% and 93% of alleles contained indels in the *TRAC* and *B2M* loci, respectively (**Figure 1B** right), both with high knockout scores.

We next analyzed the effect of the CRISPR/Cas9 editing on the T cell phenotype (**Figure 1D**) and expression of the different surface markers (**Figures 1E, F**) at different days after genome editing of T cells and CAR T cells. Some significant differences were found in the Tscm populations 4 days after the editing in the CAR population, although at day 11 these differences disappeared (**Figure 1D**) and editing seems not to affect phenotype at endpoint. In addition, no significant differences in exhaustion markers were observed (**Figure 1E**), substantiating a minimal effect of genome editing on CAR T cell fitness.

Additionally, for a more in-depth examination of the viability status of edited cells, we quantified the expression of activation induced cell death (AICD) markers, 7 days after genomic edition, and no significant differences were found in the different CAR products (**Figure 1F**), and we could conclude that there was no negative effect due to edition.

### 3.2 Safety assessment of the CRISPR/Cas9 edition strategy

**Figure 2:**
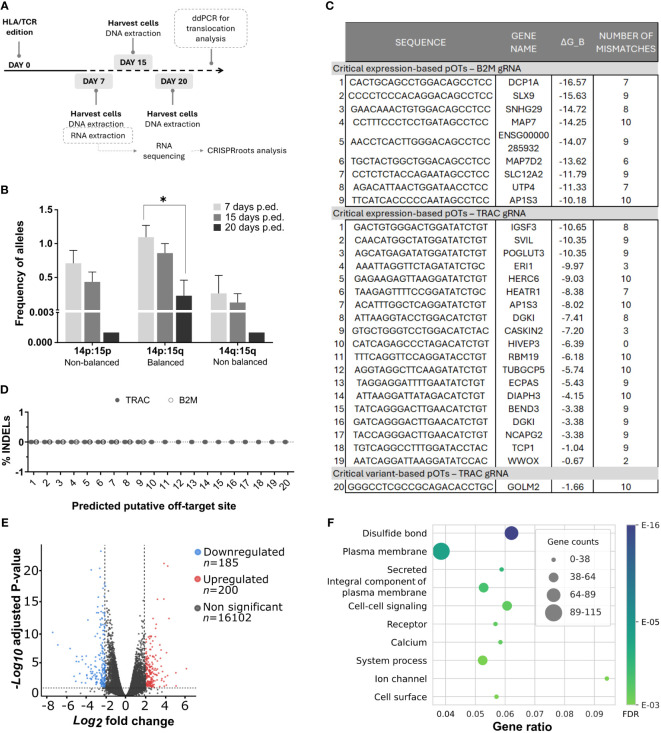
Safety analysis did not predict any important dysfunctional alterations directly caused due to simultaneous *B2M/TRAC* gene editing. **(A)** Workflow followed for the multiple safety analysis. **(B)** Graph showing alleles frequencies of different translocations found by ddPCR at day 7, 15 and 20 post editing. N=3. **(C)** Table showing the critical potential off-targets derived from the CRISPRroots analysis. ΔG_B represents binding energy obtained as in the model described in (Alkan et al., 2018). Binding energy model: ΔG_B_ = δ_PAM_(ΔG_H_ − ΔG_O_ − ΔG_U_), with ΔG_H_ the RNA-DNA hybridization energy, weighted using positional weights measuring Cas9 influence, ΔG_O_ the DNA-DNA binding energy, ΔG_U_ the spacer RNA self-folding energy (minimum free energy), and δ_PAM_ is a PAM correcting factor. **(D)** Putative off-target sites described in the table were sequenced and INDELs were analyzed by ICE Synthego web tool. N=4. **(E)** Volcano plot showing differentially expressed genes (DEGs) in our double edited cells. The plot showcases the fold change of gene expression on the x-axis, with values indicating upregulation to the right and downregulation to the left. The statistical significance, represented as -log10 (p-value), is plotted on the y-axis. In this plot, genes that are downregulated in the double edited cells are highlighted in blue, while upregulated genes are shown in red. Genes with non-significant changes in expression are displayed in grey. In CRISPRroots DEGs are genes with average DESeq2 normalized reads > 10, absolute log2 fold change > 0.5, and Benjamini-Hochberg adjusted Wald test P-value< 0.05. While these thresholds attempt to maximize the recall of DEGs, for the overrepresentation analysis DEGs they were further filtered for absolute log2 fold change ≥ 2 to better isolate the most radical changes. **(F)** The figure illustrates the relationship between gene ratios and their associated functionalities within the dataset. The x-axis represents the gene ratios, which correspond to the fraction of differentially expressed genes among all expressed genes with a shared annotation, while the y-axis showcases the corresponding functional categories or annotations. Friedman test was performed in comparisons. *p< 0.05. Non-significant results were not labelled in the graphs.

To address potential safety risks associated with our genome editing procedure, we analyzed the presence of relevant undesired events such as off-target cuts and chromosomal translocations. For such issue, we followed the workflow represented In **Figure 2A**. After CRISPR/Cas9 edition, we harvested cells at day 7, 15 and 20. For translocation analysis, we extracted DNA from these cells and performed a ddPCR, described below. For off-target analysis, RNA was isolated and sequenced from cells harvested at day 7.

To identify potential translocations as a consequence of simultaneous cuts in *TRAC* and *B2M* loci, we generated *in silico* all 8 possible rearrangements between cleaved on-targets (**Supplementary Figure 1**) and designed compatible primers and probes flanking the targeted loci. Initially, we identified by conventional PCR three occurring translocations in different donor samples: one balanced translocation Chr14p:Chr15q; and 2 non-balanced Chr14p:Chr15p and Chr14q:Chr15q harboring either two or no centromeres, respectively. As expected (45), non-balanced translocations were reduced after 20 days of culture, although balanced translocations were diluted too in the same period (**Figure 2B**).

We conducted RNA sequencing on edited and non-edited T cells for a comprehensive analysis of the impact of simultaneous *TRAC* and *B2M* genome editing and processed the data through the CRISPRroots pipeline (22). CRISPRroots identifies two classes of potential off-targets (pOTs) for each sgRNA: 1) expression-based pOTs, which are searched in a given reference genome allowing up to 6 mismatches or wobble base-pairs in the binding with the sgRNA and are sorted based on their overlap with differentially expressed genes and their binding propensity with the sgRNA; and 2) variant-based pOTs, in which somatic variants between edited and non-edited cells are linked to pOTs, which are then sorted by binding propensity.

For the B2M and TRAC sgRNAs CRISPRroots identified, respectively, 9 and 19 expression-based pOTs rated as “critical”, meaning that these pOTs are linked to downregulated genes and do not present mismatches to the gRNA in a seed region of 10 PAM-proximal nucleotides. The pipeline also identified one variant-based pOT for the TRAC sgRNA rated as “critical”, meaning it is located near a somatic variant discovered in the RNA sequencing data and does not present seed mismatches to the gRNA (**Figure 2C**; **Table 1**). Although these pOTs were ranked “critical” by the CRISPRroots pipeline, the binding energy of the spacer at these sites is far higher than at the on-target and indicates poor binding stability (**Table 1**) (final outcome of the pipeline in **Supplementary Material**). We further examined these sites by Sanger sequencing and following ICE analysis and did not uncover any conclusive evidence supporting the presence of off-targets (**Figure 2D**). All together this data led us to deduce that the genes found to be differentially expressed between knockout and wildtype cells (**Figure 2E**) are likely a consequence of the targeted knockout of *B2M* and *TRAC*, rather than off-target effects.

**Table 1:** Potential off-targets (pOTs) ranked as “critical” by CRISPRroots.

COORDINATES(1-based inclusive)	OFF-TARGET+CONTEXT	PAM	BINDING_STRUCT(Q-T)	FULL MATCH-MISMATCH PATTERN	ΔG_B	N. MISMATCH/UNUSED	GENES INTERESTED
Expression-based pOTs for B2M spacer: ACTCACGCTGGATAGCCTCC on-target ΔG_B= -25.73, specificity score CRISPRspec = 7.86 (high specificity)							
chr3:53322664-53322685:+	CACTGCAGCCTGGACAGCCTCC	TGG	XXXXXXX|||||W|||||||_XXXXXXXXX|||||W|||||||	MMM||MM|||||W|||||||_MMM||MM|||||W|||||||	-16.57	7	CACNA1D
chr21:44945403-44945424:-	CCCCTCCCACAGGACAGCCTCC	TGG	XXXXXXXXX|||W|||||||_XXXXXXXXXXX|||W|||||||	M|||M|W|M|||W|||||||_M|||M|W|M|||W|||||||	-15.63	9	SLX9
chr17:16429570-16429591:-	GAACAAACTGTGGACAGCCTCC	CAG	XXXXXXXX||||W|||||||_XXXXXXXXXX||||W|||||||	||MM||MM||||W|||||||_||MM||MM||||W|||||||	-14.72	8	LRRC75A
chr6:136406447-136406468:-	CCTTTCCCTCCTGATAGCCTCC	TGG	XXXXXXXXXX||||||||||_XXXXXXXXXXXX||||||||||	MM||M|M|WM||||||||||_MM||M|M|WM||||||||||	-14.25	10	MAP7
chr10:99651259-99651280:-	AACCTCACTTGGGACAGCCTCC	CAG	XXXXXXXXX|||W|||||||_XXXXXXXXXXX|||W|||||||	M|||||MMM|||W|||||||_M|||||MMM|||W|||||||	-14.07	9	ENTPD7
chrX:20097313-20097334:+	TGCTACTGGCTGGACAGCCTCC	TGA	XXXXXX||||||W|||||||_XXXXXXXX||||||W|||||||	MMM|MM||||||W|||||||_MMM|MM||||||W|||||||	-13.62	6	MAP7D2
chr5:128098860-128098881:+	CCTCTCTACCAGAATAGCCTCC	AAG	XXXXXXXXX|W|||||||||_XXXXXXXXXXX|W|||||||||	M|||MMM|M|W|||||||||_M|||MMM|M|W|||||||||	-11.79	9	SLC12A2
chr16:69161920-69161941:-	AGACATTAACTGGATAACCTCC	AGA	XXXXXXX|||||||W|||||_XXXXXXXXX|||||||W|||||	||MMMMW|||||||W|||||_||MMMMW|||||||W|||||	-11.33	7	UTP4
chr2:223751416-223751437:+	TTCATCACCCCCAATAGCCTCC	TGA	XXXXXXXXXXW|||||||||_XXXXXXXXXXXXW|||||||||	MM||||M|WMW|||||||||_MM||||M|WMW|||||||||	-10.18	10	AP1S3
Expression-based pOTs TRAC spacer: TCAGGGTTCTGGATATCTGT on-target ΔG_B= -21.58, specificity score CRISPRspec = 5.20 (medium specificity)							
chr1:116623693-116623714:-	GACTGTGGGACTGGATATCTGT	GAG	XXXXXXXX||||||||||||_XXXXXXXXXX||||||||||||	WMMM||MM||||||||||||_WMMM||MM||||||||||||	-10.65	8	IGSF3
chr10:29617348-29617369:-	CAACATGGCTATGGATATCTGT	AAG	XXXXXXXXX|||||||||||_XXXXXXXXXXX|||||||||||	M||M||W|M|||||||||||_M||M||W|M|||||||||||	-10.35	9	SVIL
chr11:108439331-108439352:+	AGCATGAGATATGGATATCTGT	AAG	XXXXXXXXX|||||||||||_XXXXXXXXXXX|||||||||||	WMM|W|M|M|||||||||||_WMM|W|M|M|||||||||||	-10.35	9	POGLUT3
chr8:9039809-9039830:-	AAATTAGGTTCTAGATATCTGC	CAG	XXXW||||||W||||||||W_XXXXXW||||||W||||||||W	MMMW||||||W||||||||W_MMMW||||||W||||||||W	-9.97	3	ERI1
chr4:88385686-88385707:+	GAGAAGAGTTAAGGATATCTGT	AAG	XXXXXXXXXX||||||||||_XXXXXXXXXXXX||||||||||	MM||W|||MM||||||||||_MM||W|||MM||||||||||	-9.03	10	HERC6
chr1:236562926-236562947:-	TAAGAGTTTTCCGGATATCTGC	GGA	XXXXXXX||W|||||||||W_XXXXXXXXX||W|||||||||W	MM||MM|||W|||||||||W_MM||MM|||W|||||||||W	-8.38	7	HEATR1
chr2:223689455-223689476:-	ACATTTGGCTCAGGATATCTGT	GGA	XXXXXXXXXX||||||||||_XXXXXXXXXXXX||||||||||	MMMM||W||M||||||||||_MMMM||W||M||||||||||	-8.02	10	AP1S3
chr7:137405020-137405041:+	ATTAAGGTACCTGGACATCTGT	GGA	XXXXXXXX|||||W||||||_XXXXXXXXXX|||||W||||||	|M|||MMW|||||W||||||_|M|||MMW|||||W||||||	-7.41	8	DGKI
chr17:75469014-75469035:-	GTGCTGGGTCCTGGACATCTAC	AGG	XXX||||W|||||W||||WW_XXXXX||||W|||||W||||WW	M|M||||W|||||W||||WW_M|M||||W|||||W||||WW	-7.2	3	CASKIN2
chr1:41800632-41800653:-	CATCAGAGCCCTAGACATCTGT	AGG	||||W|WW||W||W||||||_XX||||W|WW||W||W||||||	||||W|WW||W||W||||||_||||W|WW||W||W||||||	-6.39	0	HIVEP3
chr12:113874481-113874502:-	TTTCAGGTTCCAGGATACCTGT	CAG	XXXXXXXXXX|||||W||||_XXXXXXXXXXXX|||||W||||	|||||M|W|M|||||W||||_|||||M|W|M|||||W||||	-6.18	10	RBM19
chr15:23000525-23000546:+	AGGTAGGCTTCAAGATATCTGT	GGA	XXXXXXXXXXW|||||||||_XXXXXXXXXXXXW|||||||||	MM|||M|||MW|||||||||_MM|||M|||MW|||||||||	-5.74	10	TUBGCP5
chr9:111415068-111415089:-	TAGGAGGATTTTGAATATCTGT	AGA	XXXXXXXXX||W||||||||_XXXXXXXXXXX||W||||||||	MM|||W||M||W||||||||_MM|||W||M||W||||||||	-5.43	9	ECPAS
chr13:59914820-59914841:-	ATTAAGGATTATAGACATCTGT	GAG	XXXXXXXXXXW||W||||||_XXXXXXXXXXXXW||W||||||	|M|||W||M|W||W||||||_|M|||W||M|W||W||||||	-4.15	10	DIAPH3
chr6:107080973-107080994:-	TATCAGGGACTTGAACATCTGT	GGA	XXXXXXXXX||W|W||||||_XXXXXXXXXXX||W|W||||||	||||||MWM||W|W||||||_||||||MWM||W|W||||||	-3.38	9	BEND3
chr7:137402130-137402151:+	GATCAGGGACTTGAACATCTGT	AGA	XXXXXXXXX||W|W||||||_XXXXXXXXXXX||W|W||||||	||||||MWM||W|W||||||_||||||MWM||W|W||||||	-3.38	9	DGKI
chr7:158615207-158615228:-	TACCAGGGACTTGAACATCTGT	GGA	XXXXXXXXX||W|W||||||_XXXXXXXXXXX||W|W||||||	W|||||MWM||W|W||||||_W|||||MWM||W|W||||||	-3.38	9	NCAPG2
chr6:159762279-159762300:-	TGTCAGGCCTTTGGATACCTAC	AGA	XXXXXXXXX||||||W||WW_XXXXXXXXXXX||||||W||WW	|||||MW|M||||||W||WW_|||||MW|M||||||W||WW	-1.04	9	TCP1
chr16:78184267-78184288:+	AATCAGGATTAAGGATATCCAC	TGA	|||||W||MM|||||||WWW_XX|||||W||MM|||||||WWW	|||||W||MM|||||||WWW_|||||W||MM|||||||WWW	-0.67	2	WWOX
Variant-based pOTs for TRAC spacer TCAGGGTTCTGGATATCTGT on-target ΔG_B= -21.58, specificity score CRISPRspec = 5.20 (medium specificity)							
chr15:44284054-44284075:-	GGGCCTCGCCGCAGACACCTGC	AGG	XXXXXXXXXXW||W|W|||W_XXXXXXXXXXXXW||W|W|||W	M|MMM|WWMWW||W|W|||W_M|MMM|WWMWW||W|W|||W	-1.66	10	GOLM2

The table reports properties of the gRNAs used for editing and their top pOTs found by our CRISPRroots RNA-seq analysis pipeline. The first column reports the location of the pOTs in the variant-aware genome, which is a modified version of the reference genome in which variants (including indels) discovered in the non-edited cells are introduced. Positions are 1-based and include both ends. The coordinates include two nucleotides of PAM-distal context. The columns “off-target + context” and “PAM” report, respectively, the pOT sequence in 5’-3’ direction, including context, and the PAM. The column “binding struct (Q-T)” displays the most favorable, i.e. lowest free energy, potential binding pattern between the gRNA (query, Q) and the pOT site (target, T). The binding pattern is encoded as follows: X=Base not used (possible only placed at left-end); |, Base pair; W, wobble base pair; B, bulge. The binding pattern of the gRNA and of the pOT are separated by an underscore; in the absence of bulges, the pattens are identical except that the pOTs have two additional X on the left side, representing unbound context nucleotides. The pOTs and gRNA spacer are in many cases predicted to bind only partially from the PAM. Compared to the “binding struct (Q-T)” column, the column “full match-mismatch pattern” shows how the unbound nucleotides (X) would bind if the binding energy was disregarded and all nucleotides, excluding the unbound context, were used in the binding. ΔG_B_ represents the gRNA-target binding energy obtained as in the model described in (Alkan et al., 2018), in which ΔG_B_ = δ_PAM_(ΔG_H_ − ΔG_O_ − ΔG_U_), with ΔG_H_ the RNA-DNA hybridization energy, weighted using positional weights measuring Cas9 influence, ΔG_O_ the DNA-DNA binding energy, ΔG_U_ the spacer RNA self-folding energy (minimum free energy), and δ_PAM_ is a PAM correcting factor. The column “n. mismatch/unused” reports the number of nucleotides in the targets that represent a mismatch or that are not used to form the most favorable energetic binding. Last, the column “genes interested” summarizes the content of the CRISPRroots output column “genomic features” and shows the name of the genes downregulated for the expression-based pOTs or possibly affected by a somatic variant for the variant-based pOTs. For each gRNA, we report the on-target ΔG_B_ for comparison with the pOTs and the CRISPRspec gRNA specificity score from (Alkan et al., 2018), which evaluates the specificity of the gRNA to its on-target site while considering all the pOTs as alternative binding sites.

Finally, we investigated the cellular functions and components affected by the knockout of *B2M* and *TRAC* locus. We inspected annotation terms related to 385 differentially expressed genes (**Figures 2E, F**) and as expected, among the most significant annotation terms were the Gene Ontology Biological Process “Cell-cell signaling”, the Gene Ontology Cellular Components “Plasma membrane”, “Integral component of plasma membrane” and “Cell surface”, as well as the UniProt Keywords “Secreted” and “Receptor” (**Figure 2F**). Although not specific to a pathway or function, these terms align with the role of the edited genes.

### 3.3 Universal CAR T cells proved to be effective against tumoral cells

**Figure 3:**
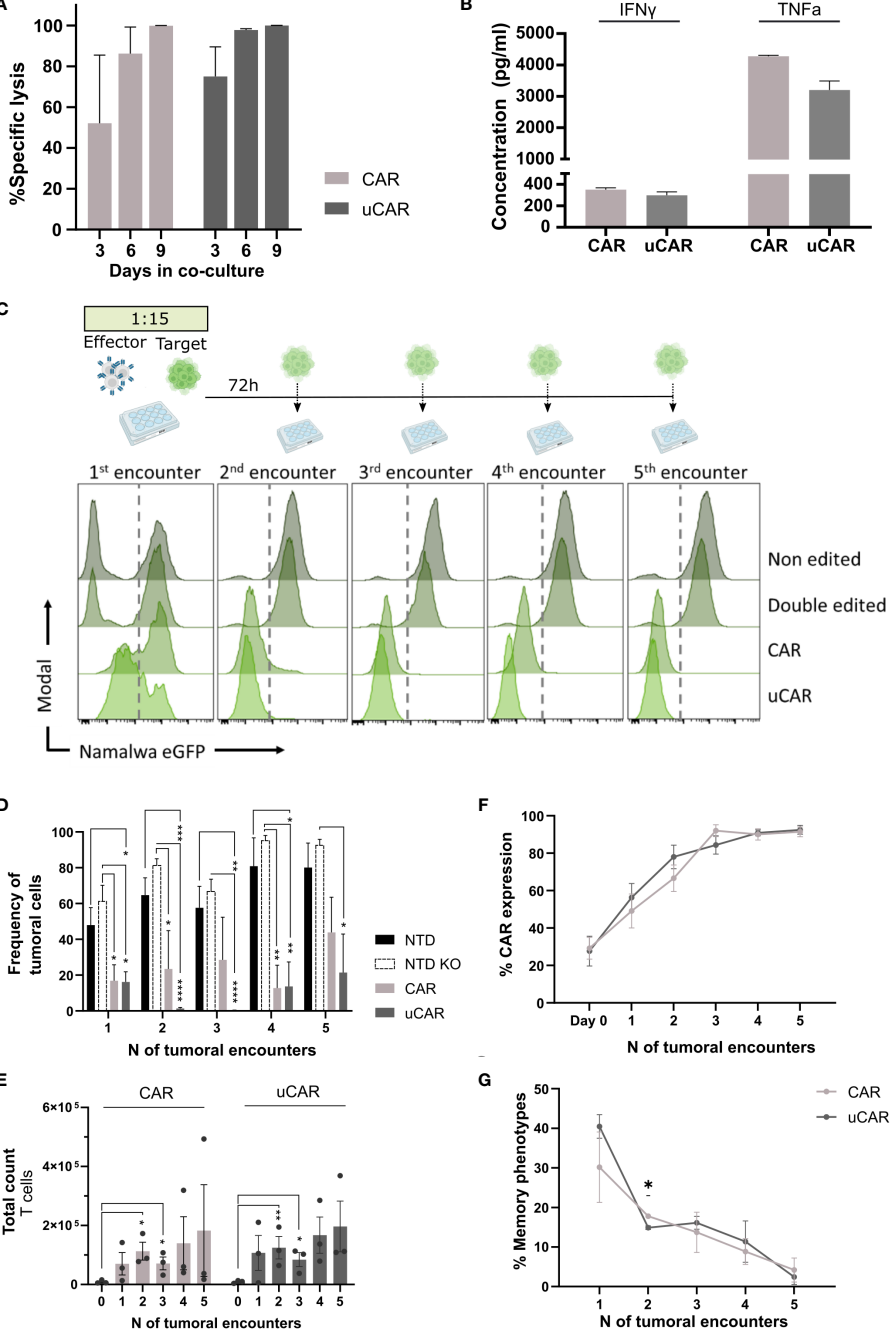
Universal CAR T cells in co-culture with CD19^+^ tumoral cells act as non-edited CAR T cells. **(A)** Graph showing percentage of specific lysis of. CAR and uCAR. This parameter was calculated considering specificity when co-culturing tumoral cells with both CD19^+^ and CD19^-^ cells and evaluating lysis in both cases. **(B)** ELISA-based quantification of IFN-γ and TNF-α secreted by effector cells after the first encounter with Namalwa cells. N=4. **(C)** Above.Timeline followed for cytotoxicity experiments. At day 0 tumoral cells are seeded with CAR T cells at ratio 15:1 respectively. Every 72h CAR T cells are rechallenged with tumoral cells. Below. Histograms resulting of cytometric analysis of the different co-culture wells showing presence of Namalwa CD19^+^ cells, tracked by FITC expression. In each histogram T cells can be tracked in the left and Namalwa in the right side (divided by a discontinuous line). **(D)** Graph representing frequency of tumoral cells in the different tumoral encounters. **(E)** T cells expansion, in terms of absolute numbers, over the tumoral rechallenges. **(F)** CAR expression in CAR and uCAR populations in co-culture with the tumoral cells. **(G)** Frequency of the sum of Tscm and Tcm after the different tumoral encounters; Friedman and Wilcoxon tests were performed. *p< 0.05, **p<0.01, ***p<0.001, ****p<0.0001. Non-significant results were not labelled in the graphs.

Having established the safety and efficacy of dual gene editing, we had to ensure that universal CAR T cells performed as well as non-edited CAR T cells, when referring to functionality. In this line, we co-cultured uCAR T cells with the Namalwa CD19+ tumoral cell line and Namalwa CD19- as control cells, in a 15:1 target to effector ratio. We evaluated specific lysis after a single encounter at different time points (**Figure 3A**) and lysis after multiples encounters (**Figures 3C, D**) of uCAR T and non-edited CAR T cells. We evaluated secretion of IFN-γ and TNF-α cytokines after the first encounter (first 72h), and we did not find any differences between pools (**Figure 3B**).

The uCAR T cells killed Namalwa cells with the same specificity at single tumoral doses (**Figure 3A**) and with the same efficacy as regular CAR T cells after several encounters (**Figures 3C, D**). In addition, we observed no statistical differences between the two CAR T products when comparing T cells expansion (**Figure 3E**), CAR expression, which is enriched from 30% to 90% (**Figure 3F**) or T cells memory phenotype (**Figure 3G**) at final point. All these data suggest that genome editing did not alter the functional potential of our uCAR T product. Moreover, in comparison to non-transduced cells (NTD) and non-transduced cells knocked out for HLA and TCR (NTD KO), the uCAR product exhibited significantly greater differences in terms of Namalwa lysis.

### 3.4 uCAR T cells showed decreased alloreactivity in an allogeneic setting

**Figure 4:**
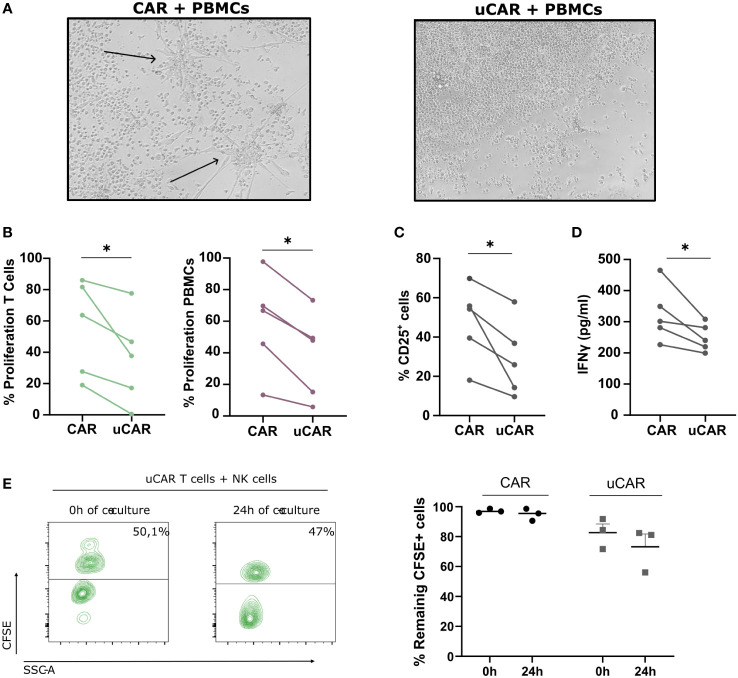
Alloreactivity Analysis of Universal CAR T Cells. **(A)** In a well with a 6-day co-culture 1:1 (T cells-PBMCs) no visible reaction is found using an optic microscope (10X). The arrows denote the differentiation signals of PBMCs visible to the naked eye. **(B)** When evaluating proliferation, based on CFSE and CTV dilution, we found a more resting state in wells where T cells did not present TCR nor HLA-I. N=5. **(C)** We measured by cytometry expression of the activation marker CD25+ in the total population and found similar results. N=5. **(D)** IFNγ liberation was measured by ELISA and represented as pg/ml. N=3. **(E)** Proportion of T cells (CFSE+) and NK cells (CFSE-) at initial (0h) and final point (24h) (left image). We represented frequency of remaining CFSE+ cells for both CAR and uCAR products. Data represented as fold change related to each donor. Wilcoxon tests were performed. *p< 0.05. No significant results were not labelled in the graphs.

To avoid any issues arising from graft-versus-host and host-versus-graft reactions, we developed a uCAR product eliminating HLA class I and TCR from T cells surface. To test successful ablation of these molecules, we established an experiment in an allogeneic setting, in which we co-cultured CFSE-labelled CAR or uCAR T cells in a 1:1 ratio with CTV-labelled PBMCs from a different healthy donor. As expected, the condition in which non-edited CAR T cells were co-cultured with PBMCs showed signs of differentiation and activation (**Figure 4A**, left image), while the response of uCAR T cells was significantly less pronounced (**Figure 4A**, right image). Also, the proliferation of both T cells (**Figure 4B**, left graph) and PBMCs (**Figure 4B**, right graph) was significantly lower in the uCAR T cell samples when compared to the regular CAR T cells.

We next analyzed CD25 expression as a marker of cell activation on both CAR T cells and PBMCs. As expected, CD25 expression was reduced in all cells present in the co-culture when incubated in the presence of uCAR T cells (**Figure 4C**). These data correlated with lower levels of IFNγ secretion (**Figure 4D**).

When analyzing NK alloreactivity, despite employing established protocols for evaluating NK cell-mediated lysis of HLA KO cells (46, 47), our investigation revealed no significant variance in lysis rates between HLA-expressing and HLA-deficient cells under identical conditions of ratio and time (**Figure 4E**). In the left panel we represented the proportion of T cells (CFSE+) versus NK cells (CFSE-) at the beginning and end of the co-cultured. T cells in both CAR and uCAR products remained practically the same after 24h of co-culture with NK cells (**Figure 4E**, right).

### 3.5 Memory uCAR T cells demonstrated an equally potent lytic capacity against tumoral cells, with a less pronounced cytokine secretion profile

**Figure 5:**
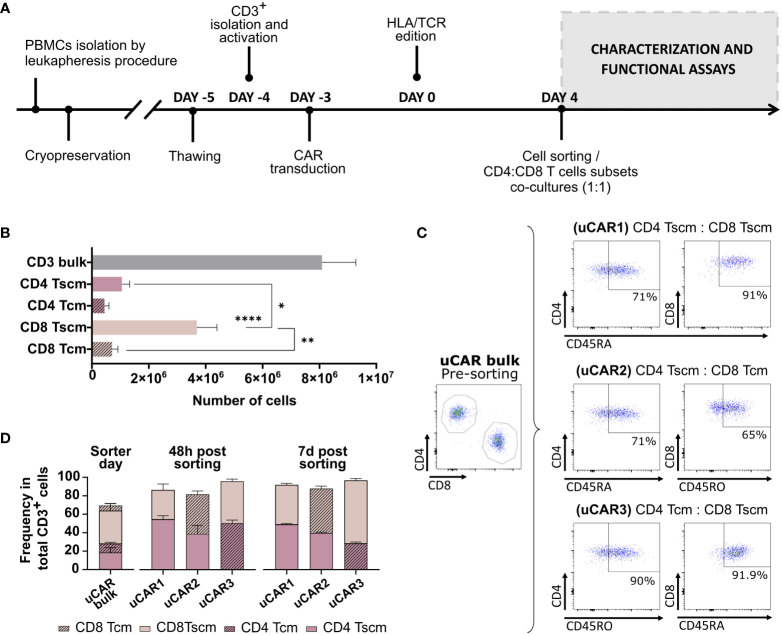
Isolation and characterization of Memory Universal CAR T cells. **(A)** Experimental designed followed during the experiment. Phenotypic and functional characterization were performed after cell sorting at day 4. **(B)** Representation of the total count of each subset after sorter of CD3^+^ total bulk. **(C)** Subsets combinations were co-cultured as shown in the figure. uCAR T cells were enriched for Tscm phenotype (CD4/8^+^ CD45RA^+^) or for Tcm phenotype (CD4/8^+^ CD45RO^+^). Percentage of each gate are representative data from all evaluated donors. **(D)** Phenotypic characterization of T cell subsets performed by flow cytometry at day 0,2 and 7 post sorting, with ratio CD4:CD8 1:1 after separation. Data are represented as mean ± SEM of three independent healthy donors. N=3. Wilcoxon and Mann-Whitney T-test are shown. *p< 0.05, **p< 0.01, ****p< 0.0001. Non-significant results were not labelled in the graphs.

Our main objective was to improve the efficacy and persistence of uCAR-T cells by selecting the less differentiated T cell populations. We, therefore, sought to identify the ideal CD4:CD8 memory T cell combination to generate an optimal uCAR T product. We optimized the experimental design shown in **Figure 5A**. To generate the different memory uCAR T products, we first isolated the following subsets: CD4+ Tscm, CD4+ Tcm, CD8+ Tscm and CD8+ Tcm, from TCR-/B2M- uCAR T cells according to the procedure described in the methodology, with CD8 Tscm being the most frequent subset after separation (**Figure 5B**). These four subpopulations were then combined in ratio of 1:1 for CD4:CD8 to generate three different combinations: CD4 Tscm: CD8 Tscm (uCAR1), CD4 Tscm: CD8 Tcm (uCAR2) and CD4 Tcm: CD8 Tscm (uCAR3) (**Figure 5C**). All three uCAR T cell products were memory-enriched comparing with the total CD3+ bulk, and the ratio was stably maintained in culture, although in the uCAR3 condition a slight increase in the ratio of CD8 Tscm to CD4 Tcm after 7 days in culture was observed (**Figure 5D**).

**Figure 6:**
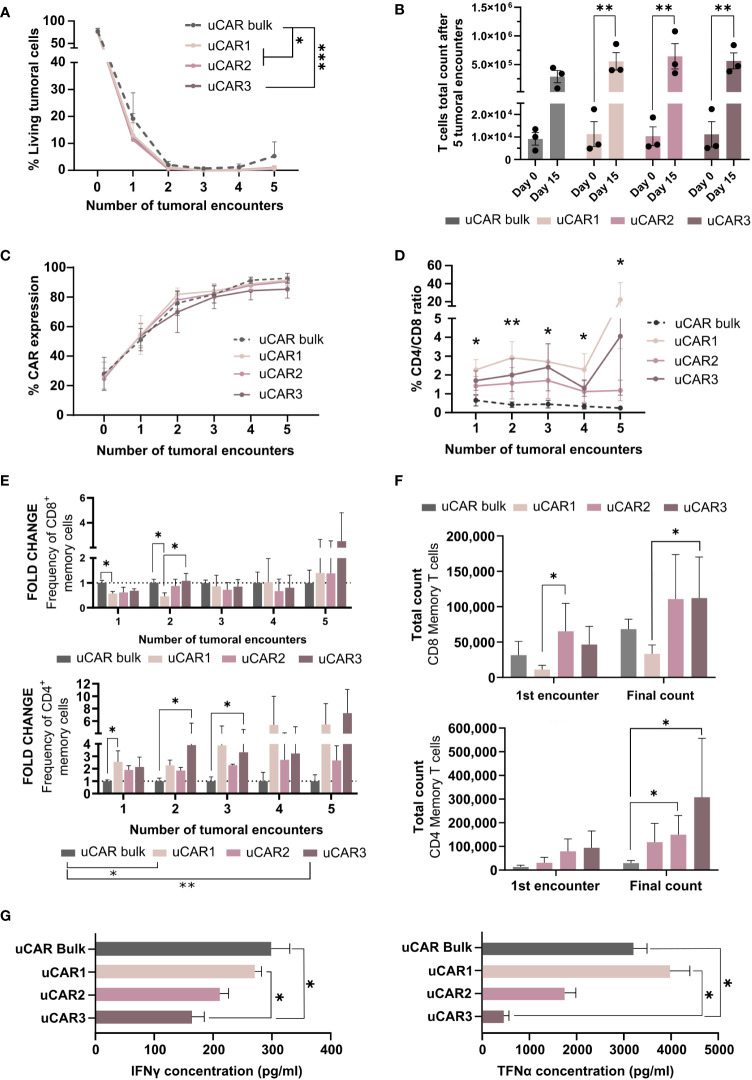
Cytolytic behavior of Memory Universal CAR T cells. **(A)** Graph showing frequency of living tumoral cells during several encounters, evaluated every 72h. **(B)** Representation of the T cells total expansion after five tumoral encounters. This data has been determined considering T cells count at each point in order to obtain the magnitude of the expansion. **(C)** CAR expression was measured by flow cytometry and represented as frequency in each well. **(D)** At each time point frequency of CD4/CD8 was evaluated by flow cytometry and represented for each set of cells as fold change related to day 0. **(E)** Representation of the summatory of Tscm and Tcm present in CD8^+^ (left) CD4^+^ (right) populations. Fold change related to uCAR population is represented. **(F)** Absolute numbers of memory T cells in each product. This data was obtained taking into account the frequency of Tscm and Tcm in each condition and T cells count in each condition. **(G)** IFN-γ (left) and TNF-α (right) cytokine quantification by ELISA assays, secreted by effector uCAR T cells after the first encounter with Namalwa cells. N=3; Friedman and Wilcoxon tests were performed. The legends show significance levels from ANOVA comparing group means overall. Specific significances at each time point are marked with asterisks directly on the graph. *p< 0.05, **p > 0.01, ***p > 0.001.

We next analyzed the anti-tumor activity of the different uCAR-T cells products confronting 15 target cells to 1 effector CAR T cell for conditions uCAR1, uCAR2 or uCAR3. Every 72 hours, the lysis activity was monitored by fluorescence via flow cytometry and new tumor cells were added (up to 5 tumor encounters). In this context, bulk and memory uCAR T performed very similarly against tumoral cells *in vitro*. Although no significant differences were found at any single time point, significant differences were found when the complete data set was compared (**Figure 6A**). NTD and NTD KO data were previously represented in **Figure 3D**.

Multiple evaluations were performed on the T cell populations of each condition. First, in order to evaluate CAR T cells persistence and survival in co-culture with tumoral cells, total cell count was determined every 72h, and we did observe that at final point, the expansion of the uCAR memory products increased significantly when compared to the beginning of the experiment, which uCAR bulk T cells did not (**Figure 6B**), this meaning that the memory uCAR products present higher expansion capacity in co-culture with tumoral cells. As expected, regardless to the combination of uCAR T cells, these T cells presented the same frequencies of CAR in their surface (**Figure 6C**).

On the other hand, despite having started the co-culture experiments at ratios of 1:1 for CD4 to CD8 for all three combinations, we observed an increase in the CD4 population, which was most pronounced in the uCAR1 combination (**Figure 6D**). When evaluating the memory compartment (%Tscm and %Tcm) in CD8+ (**Figure 6E**, left) and CD4+ (**Figure 6E**, right) populations, we did not see phenotypic improvement in CD8 cells but we did in CD4 cells, where phenotype seems clearly improved compared to the bulk product. When considering final count of memory T cells, after five tumoral encounters there were more T memory cells in the co-cultures with the uCAR T products (**Figure 6F**) which indicated persistence of this phenotype after exerting their functional activity. Additionally, to assess the cytokine secretion profile of each product, we quantified the secretion of TNFα and IFNγ inflammatory cytokines by ELISA after the first tumoral encounter (**Figure 6G**), observing a reduction in the production of both by some of the combinations.

## 4 Discussion

Currently, treatment for relapsed/refractory (R/R) B-ALL and hard-to-treat lymphomas remains a clinical unmet need. Rapid research in the CAR-T cell therapy field is paving the way for this treatment to become an important chance of immunotherapy for these patients. Although CAR-T cells have shown promising results, production of such cells can take up to 4 weeks, and disease can remarkably progress in patients during this waiting period (29, 48). Also, persistence after administration remains an important issue, as it is directly related to therapy success (29). Many studies have already suggested that the selection of specific formulations of CAR-T cells may improve future outcomes of the therapy (34, 49, 50). In this study we focused both on generating a universal product which would eliminate the waiting time for the patients to be treated, and on the selection of specific subsets of these products in order to maintain its antitumoral activity over time.

The primary objective of this study was to evaluate and optimize safer methods for the generation of CRISPR-Cas9-edited cells. As many others have accomplished along the years (51–53), we successfully eliminated TCR and HLA-I from T cells and CAR T cells, and we proved that the decrease in expression is stable in culture over time. Our editing strategy was based on the CRISPR/Cas9 system, as it holds a higher degree of adaptability and the ability to manipulate multiple genes simultaneously, making it more appropriate in comparison to alternative endonuclease-related technologies (54, 55). The concern surrounding the use of CRISPR-Cas9 in clinical settings stems from the potential genotoxicities it could induce, both at transcriptomic and genomic levels. The consideration of these potential off-target effects raises questions about the precision and safety of the editing process. Off-target events can occur when the sgRNA used for gene editing mediates binding to an undesirable site in the genome, due to the existence of sufficient homology in its sequence. It is important to carefully design the sgRNA to ensure not only a high level of editing efficiency on-target, but also to minimize the potential for unintended off-target effects. Specific tools for off-target detection have been developed to tackle such issue (22, 56–58). Utilizing the CRISPRroots pipeline (22) we found that the potential off-target sites exhibited low binding affinity with the sgRNAs employed for the double edition. Additionally, when we sequenced the top candidates proposed by CRISPRroots, we did not find generation of new insertions or deletions. Therefore, we concluded that the differential expression of genes that we found between edited and non-edited cells primarily arises from the intended editing process, rather than being attributed to off-target events. Despite we detected some alterations at the transcriptomic level, in culture we have not detected any disadvantage in our edited cells compared to the unedited ones, neither functionally nor phenotypically. On the other hand, chromosomal rearrangements are a possibility when performing dual editing with CRISPR/Cas9 and have been described in edited CAR T cells before (59). In line with other researchers (45), we did find some chromosomal translocation events at low frequencies (up to 1.5%) and they seemed to decrease over time. The loss of the rearrangements suggests that the translocations found did not confer a competitive advantage to the cells and may not be considered a concern preventing further examinations of double edited cells *in vivo*. Such aberrations could be further reduced by using, for instance, prime editing strategies for executing the editing steps (60).

The main challenge of the allogeneic approach in CAR-T therapies is the dual alloreaction risk – both by and of the infused cells in the patient. We successfully showed that by eliminating *B2M* and *TRAC* genes, alloreactive responses were reduced *in vitro*, suggesting that modifying the expression of these surface proteins could improve allogeneic CAR-T efficacy and safety. Regarding other alloreactive responses that these cells can trigger, many studies have described NK cell attacks towards HLA-I deficient cells (46, 47). NK cells play a crucial role in immune surveillance by targeting cells lacking HLA-I molecules, a condition known as “missing self-recognition”. Some of these molecules act as inhibitory ligands to NK cell receptors, so if they are not present on the cell surface, NK cells readily respond, leading to their elimination (61). However, in our *in vitro* evaluation we did not find any difference in frequency of CAR or edited uCAR cells after 24h co-culture with NKs.

Based on prior research (62, 63), it has also recently been corroborated that NK cells do not always target HLA-I deficient cells strongly (64). concluded in their research that the presence of certain HLA-II molecules may serve as ligands for NK cell receptors, which could explain why our double-negative cells for both TCR and HLA-I are not being attacked by NK cells in co-culture. However, the interaction between the NK cells and the HLA-I receptor is highly complex, and our experiment is limited, meaning it does not completely exclude the possibility that our uCARs may be lysed by NK cells *in vivo*, and further studies may be needed in the future.

However, the overriding question is to determine whether the effects of this CRISPR/Cas9 editing could be phenotypically detrimental to the cells, potentially causing the loss of less differentiated cells. In this sense, our results demonstrate no significant changes upon the memory compartment 11 days after editing, nor differences in the expression of exhaustion markers. Accordingly, the functionality of the cells was also intact, as uCAR T cells performed equally as standard CAR T cells in co-culture with tumoral cells, as others have previously proved (51, 65, 66).

In the clinical setting, it has been verified that the best outcomes have been related to a higher proportion of memory cells in the infused CAR-T product (27), and therefore some protocols are producing CAR T cells after memory cells isolation and expansion (67–69). However, some recent reports presented conflicting viewpoints regarding the impact of memory subsets on the effectiveness and functionality of CAR T cells *in vitro*. While Arcangeli et al. showed a less pronounced effector signature of memory stem cells compared to bulk CAR T cells *in vitro* (68), Meyran et al. presented a significantly higher secretion of key effector molecules and enhanced proliferative capacity in TSTEM-like CAR-T cells (34). Here we report the feasibility of isolating these cells after edition and transduction of the final product, to ensure that at the time of infusion and recognition of the tumor the memory phenotype is the predominant one. In contrast to what these and other authors had described *in vitro* (34, 68), our memory uCAR T cells presented equal lytic capacity against tumoral cells, with some evidence of improvement against uCAR bulk cells in some donors, through a less pronounced inflammatory cytokine secretion profile.

More replicates are needed for a more robust conclusion. However, the phenotype at final point indicated a trend towards an improvement in the quantity of memory uCAR T cells, when compared to the bulk, which could be hopefully related to potential future improved *in vivo* persistence of this product. Our results showed an increase in the CD4+ population after stimulation of the uCAR pool, as others have reported (34). Although many works have focused on using only the CD8 population of CAR T cells (23, 27–29), CD4 cells play an important role in tumor regression (30–33), and some papers even claim that in some settings, CD4+CAR+ T cells outcome surpasses that of CD8+CAR+ T cells (68). Meyran et al. proved that CD4 increased proliferative capacity and cytokine secretion when present in the final CAR product (34), and therefore growing evidence suggests that preserving a 1:1 ratio of CD4 to CD8 in CAR-T cells products is pivotal for long-term antitumor responses.

As it has been described by us and other authors (67, 68, 70), memory CAR T cells produce fewer inflammatory cytokines. This fact, combined with the observation that the lytic capacity is not affected, suggests a potential for reduced incidence of cytokine release syndrome (CRS) and neurotoxicity in patients in future clinical trials, so it would be a safer approach than the standard CAR therapies.

In conclusion, in this study, we successfully and safely eliminated HLA-I and TCR expression in T cells and we meticulously addressed the potential challenges associated with off-target events in CRISPR-Cas9 edited cells. By analyzing RNA-sequencing data with the CRISPRroots pipeline, we verified that potential off-target sites exhibit low binding affinity with the sgRNAs used for the double edition. Nevertheless, a significant constraint in this study arises from the need for a subsequent *in vivo* investigation, hindered by the low yield obtained from sorting assays.

Our current focus is on optimizing the purification step. While the use of the sorter has demonstrated high performance, it poses a bottleneck in our project. Cells subjected to this process fail to expand (data not shown) even after a week in optimized G-Rex culture. The inability to reach an optimal cell number for injection into mice hinders the progress towards the *in vivo* phase. Therefore, we are currently considering the use of magnetic beads for isolation, which may not compromise cellular integrity as much and allow subsequent expansion. The encouraging preliminary outcomes established a foundation for developing a protocol employing a pre-defined universal CAR T product. This, in turn, contributes to the ongoing optimization of CAR-based therapies in clinical settings.

## Data availability statement

The original contributions presented in the study are included in the article/**Supplementary Material**. Further inquiries can be directed to the corresponding author.

## Ethics statement

The studies involving human samples were approved by Hematology Department of the Hospital Universitario Reina Sofıa (Córdoba, Spain). The studies were conducted in accordance with the local legislation and institutional requirements. The participants provided their written informed consent to participate in this study.

## Author contributions

KP: Conceptualization, Investigation, Writing – original draft, Writing – review & editing, Methodology, Validation. MDCL: Conceptualization, Investigation, Writing – original draft, Writing – review & editing, Methodology, Validation. GIC: Writing – review & editing, Data curation, Software, Formal analysis. NMP: Investigation, Methodology, Writing – review & editing. FJME: Writing – review & editing, Investigation. EPS: Resources, Writing – review & editing, Methodology. MCG: Investigation, Methodology, Writing – review & editing. PJL: Investigation, Methodology, Writing – review & editing. MTM: Investigation, Methodology, Writing – review & editing. VRD: Investigation, Methodology, Writing – review & editing. ABR: Investigation, Methodology, Writing – review & editing. AML: Investigation, Methodology, Writing – review & editing. PHV: Investigation, Methodology, Writing – review & editing. CFG: Writing – review & editing, Data curation. TC: Writing – review & editing, Data curation. SES: Writing – review & editing, Data curation. JG: Writing – review & editing, Data curation. FM: Writing – original draft, Writing – review & editing, Funding acquisition, Investigation, Resources, Conceptualization. CH: Writing – original draft, Writing – review & editing, Funding acquisition, Investigation, Resources, Conceptualization. KB: Writing – original draft, Writing – review & editing, Funding acquisition, Investigation, Resources, Conceptualization. 
