# Supplementary material for: Generating universal anti-CD19 CAR T cells with a defined memory phenotype by CRISPR/Cas9 editing and safety evaluation of the transcriptome
Source: Front Immunol. 2024 May 29;15:1401683. doi: 10.3389/fimmu.2024.1401683 (PMC11167079; doi:10.3389/fimmu.2024.1401683)
Supplement: Supplementary file 1 [file DataSheet_1.docx]

Supplementary Material

# Supplementary Figures and Tables

For more information on Supplementary Material and for details on the different file types accepted, please see [here](https://www.frontiersin.org/guidelines/author-guidelines#supplementary-material).

## Supplementary Figures


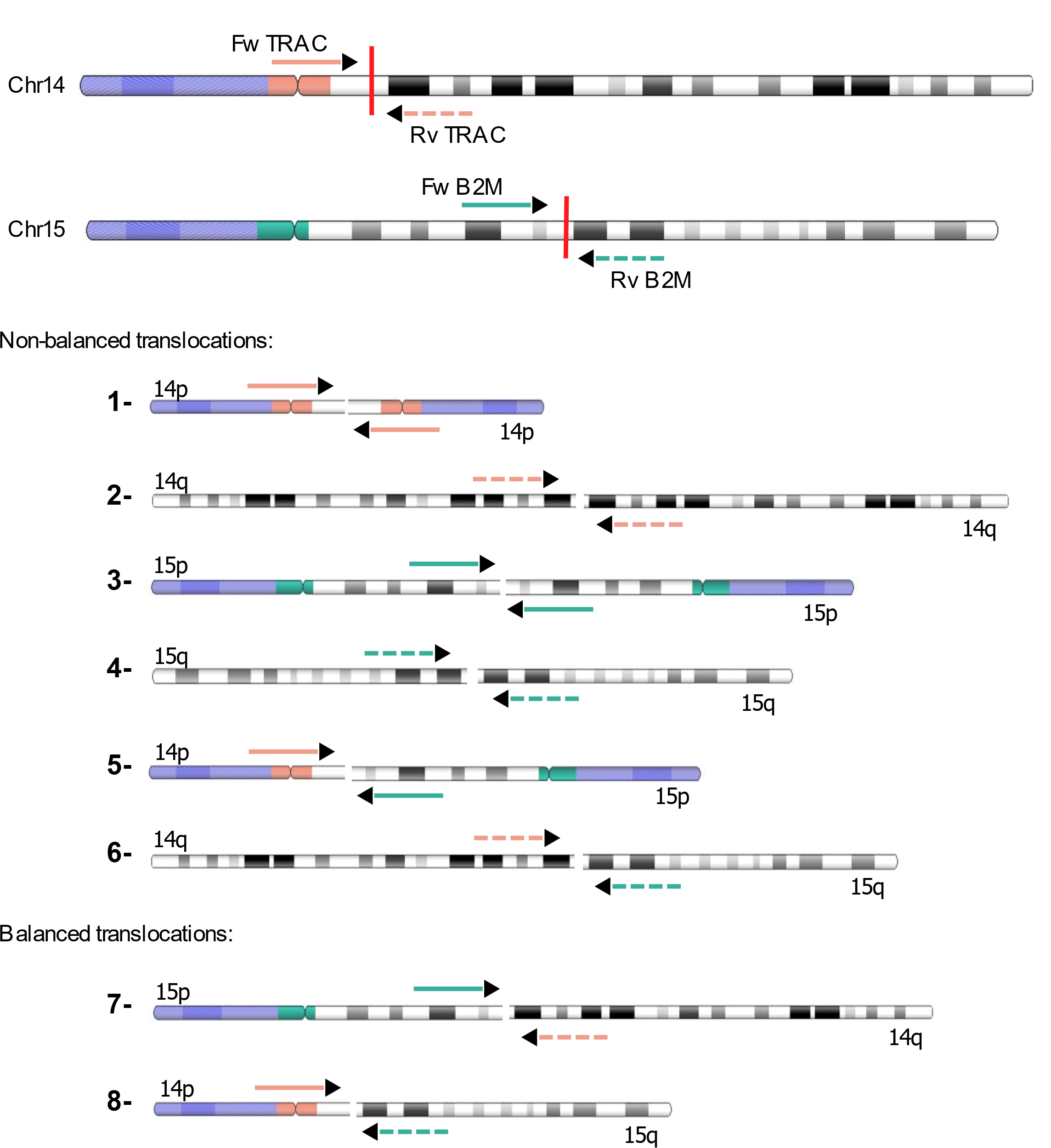


**Supplementary Figure 1. Possible occurring translocations between chromosomes 14 and 15.** Wildtype ideograms of targeted chromosomes (top). Blue lines mark the cleavage expected from the sgRNAs targeting *TRAC* and *B2M* genes. A set of compatible probes and primers were designed flanking the cutting sites. Pink and green arrows represent primers used for amplification (pink for B2M sequence, and green for TRAC sequence). Putative resulting translocations (bottom) are enumerated from 1 to 8. Rearrangements 1-6 are aberrant as they present non or more than one centromere, while rearrangements 7-8 present one in their structure.

| T Cells Phenotypic Charactarization  Allogeneic, Exhaustion and Cytotoxic assays | | | |
| --- | --- | --- | --- |
| CD3 | PerCP-Cy5.5 | clone OKT3 | Invitrogen |
| CD4 | EFluor450 | clone RPA-T4 | Invitrogen |
| CD8 | eFluor506 | clone RPA-T8 | Invitrogen |
| CD45RA | FITC | clone HI100 | Invitrogen |
| CCR7 | PE | clone 3D12 | Invitrogen |
| CD2 | PerCP-Cy5.5 | clone RPA-2.10 | BD Biosciences |
| CD25 | APC | clone CD25-4E3 | Invitrogen |
| HLA-ABC | APC | clone W6/32 | Invitrogen |
| EGFR | PE | clone AY13 | Biolegend |
| TIM3 | PE-Cya7 | clone F38-2E2 | Invitrogen |
| PD1 | PE | clone eBioJ105 | Invitrogen |
| LAG3 | eFluor506 | clone 3DS223H | Invitrogen |
| Bulk and isolated subsets phenotype monitoring and cell sorting markers | | | |
| CD3 | APC-H7 | clone SK7 | BD Pharmigen |
| CD4 | BV605 | clone RPA-T4 | BD Biosciences |
| CD8 | BB515 | clone RPA-T8 | BD Horizon |
| CD45RA | APC | clone HI100 | BD Pharmigen |
| CD45RO | PE | clone UCHL1 | BD Pharmigen |
| CCR7 | BV421 | clone 2L1A | BD Pharmigen |
| CD95 | R718 | clone G46-2.6 | BD Biosciences |

**Supplementary Table 1.** Description of monoclonal antibodies used in flow cytometry: Identification, fluorochrome conjugate, clone and manufacturer.
